# Challenging the Negative Perception of Lithium and Optimizing Its Long-Term Administration

**DOI:** 10.3389/fnmol.2018.00349

**Published:** 2018-10-02

**Authors:** Janusz K. Rybakowski

**Affiliations:** Department of Adult Psychiatry, Poznan University of Medical Sciences, Poznan, Poland

**Keywords:** lithium, bipolar, long-term treatment, thyroid, kidney, cognition

## Abstract

The use of lithium for the prevention of recurrences in mood disorders has a 55-year history. Nowadays, lithium is universally accepted as the first-choice mood-stabilizer (MS) for maintenance treatment of bipolar disorder. In addition to its mood-stabilizing properties, lithium exerts anti-suicidal, immunomodulatory and neuroprotective action which may further substantiate its clinical usefulness. Despite these facts, the use of lithium in mood disorders has been greatly underutilized. The reasons include the introduction and promoting other MS as well as a perception of lithium as a “toxic drug” due to its side effects, mainly thyroid, renal and cognitive disturbances. The trends in lithium prescription in recent decades show relative stability or a decline at the expense of other mood-stabilizing drugs, both first generation (valproate) and second generation (olanzapine, quetiapine, lamotrigine). In this review article, the negative perception of lithium by some clinicians will be challenged. First, the data showing lithium superiority over other MS will be presented. Second, the lithium-induced side effects which can make a challenge for a more frequent application of this drug will be delineated, and their proper management described. Finally, an issue of benefits of long-term administration of lithium will be discussed, including the phenomenon of the “excellent lithium responders” (ER) as well as a subject of starting lithium prophylaxis early in the course of the illness. This review article is based on the 47-year experience with lithium therapy by the author of the article.

## Introduction

In this year, the 55th anniversary of the first publication on lithium prophylactic effect in mood disorders (Hartigan, 1963) is observed. In 1970–1973, the results of eight placebo-controlled studies were published on the prophylactic lithium efficacy, using mostly a discontinuation design. The average of 30% of lithium-treated patients had relapses of mood episodes compared with the mean 70% of patients receiving placebo (Schou and Thompsen, 1976). Later, such outcomes were questioned pointing at the inappropriate methodology of the research (Moncrieff, 1997). Nonetheless, in the 21st century, the lithium prophylactic efficacy was abundantly demonstrated in three meta-analyses (Geddes et al., 2004; Nivoli et al., 2010; Severus et al., 2014).

Therefore, lithium has nowadays been universally accepted as the first-choice mood-stabilizer for maintenance treatment of bipolar disorder. The drug can also be useful in the treatment of acute episodes, as the main augmenting strategy for antidepressants in treatment-resistant depression (Crossley and Bauer, 2007). Also, in contrast to other mood stabilizers (MS), lithium possesses many distinctive biological effects. It exerts anti-suicidal action, possesses immunomodulatory features and, over the last two decades, the neurotrophic and neuroprotective properties of lithium have been demonstrated. The latter effect is the main topic of these articles’ collection.

Despite such hard evidence for efficacy and favorable effects, the administration of lithium in bipolar illness is greatly underutilized. The reasons for this are multifold. One of them has been the introduction and active promoting of other MS. They can be divided into first-generation MS, introduced in 1960–1970 (aside from lithium, valproates and carbamazepine) and second-generation MS, introduced since second half of 1990s, such as atypical antipsychotics (clozapine, olanzapine, quetiapine, aripiprazole, and risperidone), and anticonvulsant lamotrigine (Rybakowski, 2007, 2018). Other explanation for underutilization of lithium can be a perception of lithium as a “toxic drug” due to its side effects, mainly thyroid, renal and cognitive disturbances. This belief has been widespread among physicians of various specialties but can also be held by some psychiatrists. The trends for decreased application of lithium can also be reflected in the prescription of this drug which, in recent decades, shows relative stability or a decline at the expense of other mood-stabilizing drugs both first (valproate) and second generation (olanzapine, quetiapine, lamotrigine).

In his recent article, the eminent specialist on bipolar disorder, Post (2018) deplores that lithium is underutilized in the USA, even to a greater extent than in European countries. He points to the multiple assets of lithium beyond its antimanic and prophylactic effect, such as antidepressant action, suicide prevention, pro-cognitive and anti-dementia effects as well as diminishing the frequency of a few medical conditions. He argues that the fear of lithium side-effects such as progressive renal impairment can be exaggerated, and also points at the purposefulness of the early start of the long-term prophylaxis with lithium, e.g., following the first episode of mania.

In this review article, the main factors underlying negative perception of lithium in the clinic will be challenged. First, the data showing lithium superiority over other MS will be presented. Second, the lithium-induced side effects which can make a hindrance for a more extensive application of this drug will be delineated, and their proper management described. Finally, an issue of benefits of starting lithium early and its long-term administration will be discussed including the phenomenon of the “excellent lithium responders” (ER). This review is based on the 47-year experience with lithium therapy by the author of the article.

## Trends in Prescriptions of Lithium vs. Other Mood Stabilizers

Insufficient usage of lithium can be reflected by trends in the prescription of MS in recent decades. Baldessarini et al. (2007) in 7,760 American persons with bipolar disorder evaluated their prescriptions during 2002–2003. They found that lithium was prescribed to 7% of such patients while mood-stabilizing anticonvulsants to 17%, and antipsychotics to 11% of them. Hayes et al. (2011) assessed prescribing patterns of antipsychotics and MS in primary care in 4,700 bipolar patients in the United Kingdom between 1995 and 2009. Generally, the proportion of bipolar patients offered treatment increased nearly two-fold between 1995 (40.6%) and 2009 (78.5%). During this period, the prescription of lithium raised from 22.5% to 29.3%, that of valproate from zero to 22.7%, carbamazepine from 6.5% to 7.3%, lamotrigine from zero to 6.2% and second-generation antipsychotics from zero to 35% (most frequently used were olanzapine—18%, and quetiapine—6%). Stephenson et al. (2013) analyzed the use of psychotropic drugs in Australia over a period of 2000–2011. In 2011, MS such as lithium, valproates, carbamazepine and lamotrigine accounted for 5.8% of total psychotropic defined daily doses. From 2000–2011, lithium prescription remained stable while valproate and lamotrigine markedly increased. Parabiaghi et al. (2015) assessed lithium use during a period of 2000–2010 in a region of Italy. They found that the utilization of lithium increased in 2000–2002 by 8%, decreased in 2002–2006 by 13%, and again increased in 2006–2010 by 11%.

Bramness et al. (2009) compared the use of lithium in Scandinavian countries using the country prescription data of Denmark, Norway and Sweden, for a period July 2005–June 2006. They found that 0.17, 0.21, and 0.25% of the respective population of Denmark, Norway, and Sweden, had at least one lithium prescription during this time. Recently, more specific data of MS prescription in Denmark and Sweden have been available. Kessing et al. (2016) assessed all prescription data for 3,205 Danish patients diagnosed as mania or bipolar disorder in the decade 2000–2011. They found that the rate of lithium prescription during this period diminished from 41.1% to 34%, and, in 2011, this rate was exceeded by lamotrigine (increase from 3.4% to 42.1%), and by quetiapine (the increase from 0% to 39.5%). In the same period, the prescription of valproate increased from 6.9% to 14.4%, olanzapine from 8.7% to 14.3%, and aripiprazole from 0% to 10.5%. Karanti et al. (2016) who studied changes in mood stabilizer prescription for bipolar disorder in Sweden during 2007–2013 found that lithium use decreased during the study period from 51% to 41%, with concomitant increase of lamotrigine (from 25% to 33%) and quetiapine (from 9% to 25%). The use of valproate decreased (from 18% to 14%) as well as that of olanzapine (from 21% to 17%).

Recently, an attempt has also been made to study the prescription of lithium in Poland, the home country of the author of this review. During 2004–2010, the prescription of lithium in Poland rose by 4% while in 2010–2017, this increase amounted to 16%. However, in the second half of 2017, the prescriptions of lithium for bipolar disorder were surpassed 2.9-fold by valproate, 2.1-fold by quetiapine, 1.9-fold by olanzapine and 1.8-fold by lamotrigine (Rybakowski and Checinska, in press).

## Clinical Efficacy of Lithium vs. Other Mood Stabilizers

The prophylactic effectiveness of lithium in bipolar disorder seems nowadays beyond any doubts as it has been amply demonstrated in three meta-analyses performed in the 21st century. In the first study, Geddes et al. (2004) included five randomized controlled trials of total 770 patients showing that lithium was significantly more effective than a placebo in preventing all affective relapses, being slightly better against manic than against depressive recurrences. In the second study, Nivoli et al. (2010) analyzed long-term controlled trials lasting at least half a year, including 1,561 patients, of whom 534 were receiving lithium. They showed that recent data rate the lithium prophylaxis as more effective against manic than against depressive relapses while previous research had suggested the nearly equal effectiveness of lithium against both mania and depression. The most recent meta-analysis has been performed by Severus et al. (2014). The authors included seven trials (1,580 patients) comparing lithium with placebo. They concluded that lithium was significantly superior to placebo in preventing any mood episodes and manic episodes. In some analyses, lithium was also better than placebo in preventing depressive episodes.

The pivotal trials comparing lithium with other first-generation MS (carbamazepine and valproates) were the MAP (Multicenter study of long-term treatment of Affective and schizoaffective Psychoses) and BALANCE (Bipolar Affective disorder Lithium/ANtiConvulsant Evaluation). In the MAP study, the recurrences on lithium or carbamazepine were compared in 171 bipolar patients, during 2.5 years of observation. In patients with bipolar disorder, type I (*n* = 114), lithium was better than carbamazepine. On the other hand, carbamazepine had similar efficacy in subjects with bipolar illness, type II or the type not defined (*n* = 57). In a subgroup of bipolar subjects not experiencing mood-incongruent delusions and lacking psychiatric comorbidity (*n* = 67), treatment with lithium resulted in fewer number of hospitalization compared with carbamazepine (26% vs. 62%). On the other hand, in the remaining patients (*n* = 104), a trend for a better effect of carbamazepine was observed. In the BALANCE study Kleindienst and Greil (2000), 330 bipolar patients were randomized to lithium (*n* = 110) or valproate (*n* = 110) monotherapy, or both drugs in combination (*n* = 110) and studied in an open-label design. During 24-month follow-up, 59 (54%) subjects from the combined lithium-valproate group, 65 (59%) from the lithium monotherapy, and 76 (69%) from the valproate monotherapy experienced a recurrence of a manic or depressive episode. The hazard ratio for such a primary outcome for lithium vs. valproate was 0.71 (*p* = 0.047) showing the prophylactic superiority of lithium (Geddes et al., 2010).

Goodwin et al. (2004) compared the prophylactic efficacy of lithium vs. lamotrigine, the anticonvulsant regarded as a second-generation mood stabilizer, in three groups of bipolar patients receiving lithium (*n* = 167), lamotrigine (*n* = 280) or placebo (*n* = 191), in a double-blind manner for 18 months. They found that both lithium and lamotrigine were better than placebo where time to occurrence of any affective episode was taken into account. However, lithium was better than placebo for prevention of mania and lamotrigine performed better for prevention of depression. In Severus et al. (2014) meta-analysis, comparing lithium with anticonvulsants (valproate, carbamazepine, lamotrigine), including 1,305 participants, it was found that lithium was better as far as prevention of manic episodes was concerned. On the other hand, lithium was not better than anticonvulsants for prevention of depression.

A comparison between lithium and another second-generation mood stabilizer, quetiapine, was performed in the framework of the Bipolar CHOICE (The Clinical and Health Outcomes Initiative in Comparative Effectiveness for Bipolar Disorder) project. The results of the 6-month multicenter study in which lithium (*n* = 240) and quetiapine (*n* = 242) were used in typical clinical practice settings, with adjunctive personalized treatment, did not reveal significant efficacy differences between these drugs (Nierenberg et al., 2016). In their review article, Ketter et al. (2016) suggest a comparable efficacy of lithium and quetiapine in the treatment of acute episodes of bipolar disorder and its prophylaxis, and a significantly better effectiveness of combination therapy than either agent alone.

Recently, Kessing et al. (2018) analyzed observational studies comparing monotherapy with lithium and monotherapy with a different mood stabilizer. The results showed that prophylactic lithium monotherapy was more effective compared to monotherapy with such MS as valproate, lamotrigine, olanzapine, and quetiapine. The authors concluded that, in real life, prophylactic lithium monotherapy is superior to such monotherapy with other most frequently used MS.

## Distinctive Properties of Lithium vs. Other Mood Stabilizers

Many research have shown the greatest anti-suicidal properties of lithium among all MS. As the mortality of bipolar patients (Lewitzka et al., 2015) is 2–3 times that of the general population, mainly due to suicides, Müller-Oerlinghausen et al. (1992) in 827 patients with bipolar and schizoaffective disorder given lithium treatment for more than 6 months observed that the mortality of these patients did not differ significantly from that of the general population. A recent meta-analysis performed by Cipriani et al. (2013) with 6,674 patients concluded that lithium was significantly better than placebo in reducing the number of suicides (odds ratio 0.13) and deaths from any cause (OR = 0.38). In recurrent depression, lithium was also connected with a diminished risk of suicide (OR = 0.36) and the number of total deaths (OR = 0.13) when compared with placebo. In these aspects, lithium was mostly superior to other MS or antidepressants. Of special interest are also recent data on a negative correlation between suicides and lithium concentrations in drinking water (Kapusta et al., 2011; Blüml et al., 2013).

Lithium can exert antiviral effect against herpes viruses and also immunomodulatory actions. In our study, it was demonstrated that long-term treatment with lithium resulted in disappearance or greatly diminished recurrences of labial herpes (Rybakowski and Amsterdam, 1991). Lithium can also mitigate the immune-endocrine component of the pathogenesis of bipolar disorder, such as acute phase reaction, production of pro-inflammatory cytokines and excessive activation of the hypothalamic-pituitary-adrenal axis (Rybakowski, 2000). Recently, we examined the impact of longitudinal lithium administration on very small embryonic-like stem cells (VSELs) and the mRNA expression of pluripotency and glial markers, in peripheral blood. Bipolar subjects not treated with lithium, had a higher number of VSELs, paralleling the illness’ duration, and increased expression of markers, compared to matched healthy subjects. Patients treated with lithium had a lower number of VSELs and lower expression of some markers than those not treated with lithium. We suggest that lithium can alleviate excessive regenerative and inflammatory processes in bipolar disorder (Ferensztajn-Rochowiak et al., 2018).

The neurotrophic and neuroprotective effects of lithium which make the main topic of these articles’ collection were reviewed in a recent article (Rybakowski et al., 2018). The evidence for these has gradually increased, and they have been suggested as contributing to the therapeutic mechanisms of lithium in mood disorders. Lithium can produce an increase in the volume of some brain structures and such an effect in patients treated with other MS has not been observed (Germaná et al., 2010; Lyoo et al., 2010). Neuroprotective properties of lithium designate this drug as a potential treatment modality in neurodegenerative conditions. Epidemiological research point to a possibility of diminished risk of dementia connected with lithium use, both in the general population and in patients with bipolar disorders. Such a relationship was exclusive to lithium, while subjects receiving anticonvulsants, antidepressants or antipsychotics had the risk of dementia elevated, paralleling the duration of the drug’s administration (Kessing et al., 2008, 2010).

## The Adverse Effects of Long-Term Lithium Treatment and Their Management

The issue of lithium side effects and toxicity as well as their prevalence and management strategies was recently reviewed by Gitlin (2016). It seems that the adverse effects of lithium perceived as posing a significant challenge for its long-term administration include mostly thyroid, renal and cognitive abnormalities, and they will be discussed here. In bipolar disorder, the thyroid function should be examined in the context of a role of the thyroid gland and the hypothalamic-pituitary-thyroid axis in the pathophysiology of this illness. The most frequent side effects associated with longitudinal lithium administration are goiter and hypothyroidism (Kraszewska et al., 2014). We studied 137 patients with bipolar disorder, including 98 subjects (30 male, 68 female) receiving lithium for at least 3 years (mean 19 + 10 years) and 39 subjects (12 male, 27 female) never receiving lithium, The groups were matched for age and the duration of illness. The concentration of the thyroid-stimulating hormone (TSH) and the volume of the thyroid gland was significantly higher in patients receiving lithium. However, the frequency of hypothyroidism in the course of the illness was similar in both groups (24% vs. 18%). In both lithium-treated and lithium non-treated patients the prevalence of hypothyroidism was higher in women than in men (32% vs. 7% and 22% vs. 8%, respectively) and subjects with hypothyroidism were successfully treated with levothyroxine (Kraszewska, 2018). In another study, we showed that patients who have been taking lithium for 10–20 years had similar indexes of thyroid function as the patients in which the drug was given for 20 years or more (Kraszewska et al., 2015).

The most common renal adverse effect of lithium is a decrease of renal concentrating capacity, which may appear already after a few weeks of lithium use. This side-effect can alleviate after reducing lithium dose, can be treated with amiloride and disappears after lithium discontinuation. However, the more serious concern is a possibility of lithium-induced interstitial nephropathy which can develop after 10–20 years of treatment and leads to increased creatinine concentration and a decreased glomerular filtration rate (GFR). In a recent study, Tondo et al. (2017) analyzed data from 312 patients with bipolar disorder, coming from 12 participating centers. The patients, with the mean age of 56 (range 20–89) years have received lithium carbonate for 8–48 (mean 18) years. A lowering of GFR by about 30% more than could be associated with aging, connected with longitudinal lithium administration, was observed. Nearly 1/3 of subjects had the value of GFR <60 mL/min/1.73 m^2^ more than once, more frequently after ≥15 years of lithium administration, and after 55 years of age, and 18.1% of patients had that value more than twice. However, no case of the end-stage renal failure was detected. The lowering of GFR amounted to 0.71% with each year of age and 0.92% with each year of lithium administration. Risk factors for declining GFR included longer lithium treatment, higher serum lithium concentration, older age, and medical comorbidity. A decrease of GFR was also connected with its lower initial value and with beginning treatment with lithium after 40 years of age.

In a small fraction of patients receiving long-term lithium administration, progressive renal damage may occur. Such a situation frequently fosters discontinuation of lithium and replacing with other MS. However, a decision about stopping lithium should be taken with caution, especially in good responders, since other MS may not be equally efficacious. This may result in a high risk of relapse of the illness and a further treatment-resistant course. We assessed kidney function during a 5-year follow-up in four good responders to lithium (three males and one female, aged 67–69 years and treated with lithium for 27 ± 9 years) in which the GFR was lower than 50 ml/min/1.73 m^2^. During the 5-year follow-up period, in three patients with the initial GRF between 47 and 48 ml/min/1.73 m^2^, the kidney parameters such as GFR, serum creatinine, and urine specific gravity did not show significant changes, and the patients continued lithium treatment as previously. In the patient, having GFR of 32 ml/min/1.73 m^2^), in whom GFR decreased by 14%, and serum creatinine increased by 10%, we reduced the lithium dose and arranged systematic nephrological consultations. Therefore, in good long-term lithium responders having the GFR not much lower than 50 ml/min/1.73 m^2^, we suggest continuing lithium with a yearly check on kidney parameters, and in those with a much lower GFR, a reduction of lithium dose, and nephrological observation along with more frequent monitoring would be recommended (Abramowicz et al., 2017).

Some clinicians believe that lithium use can result in an impairment of cognition. A meta-analysis of bipolar patients treated with lithium compared with patients not receiving the drug shows a moderately unfavorable effect of this drug on cognitive functions (Wingo et al., 2009). However, in experimental studies, the enhancement of learning and memory by lithium has been shown (Yazlovitskaya et al., 2006; Nocjar et al., 2007; Zhang et al., 2012). The research in Poznan demonstrated that the efficacy of lithium prophylaxis could determine the status of cognitive functions. The Wisconsin Card Sorting Test (WCST) was applied to 30 patients, including excellent lithium responders, partial responders, and non-responders. Non-responders to lithium performed significantly worse than other lithium-treated patients and healthy controls on most domains of the WCST. (Rybakowski et al., 2009). The objective of the second study was to assess the performance in neuropsychological tests from the CANTAB battery which measure spatial working memory and sustained attention, in ER (*n* = 13), compared with patients where the effect of lithium was not perfect (*n* = 47), and with healthy persons, age- and gender-matched (*n* = 60). ER performed better on all neuro-psychological tests than the remaining lithium patients, but the results were no different from those of healthy control subjects (Rybakowski and Suwalska, 2010).

Recently, Bersani et al. (2016) compared 15 bipolar patients during euthymia, treated with lithium for more than a year, 15 matched patients receiving other MS, and 15 matched control persons, using the Cambridge Neuropsychological Test Automated Battery. They found that bipolar patients had significant deficiences in visual memory and executive functions. However, only patients receiving MS other than lithium but not those on lithium presented a deterioration of visual memory.

In the recent review of lithium effect on neurocognitive functions (Rybakowski, 2016), an attempt was made to delineate possible mechanisms promoting a favorable effect of lithium on cognition. The prevention of mood episodes can be a leading component. In bipolar subjects, a relationship was found between cognitive deficiency, and a higher number and greater severity of mood episodes (Martínez-Arán et al., 2004). The neurobiological components could be connected with the neurotrophic and neuroprotective effects of lithium, with the enhancement of the brain-derived neurotrophic factor (BDNF) system and inhibition of GSK-3 as the most important processes (Quiroz et al., 2010). In bipolar patients, infection with herpes simplex virus type 1 (HSV-1) correlated with the features of impaired cognition such as e.g., decreased immediate verbal memory (Dickerson et al., 2004), while we found that long-term lithium administration can cause a total remission or a great decrease in the recurrences of labial herpes (HSV-1; Rybakowski and Amsterdam, 1991).

The adverse effect of lithium on cognition and probably on many other systems can be minimized by obtaining appropriate, but not too high, lithium concentration. In our patients, where some of them had been receiving lithium for 40 years or more, we have adhered to the Mogens Schou’s guidance for prophylactic lithium, put forward nearly three decades ago (Schou, 1981), keeping the serum lithium concentration between 0.5 mmol/l and 0.8 mmol/l. The average lithium level in our cognition research was 0.65 mmol/l and, when required, the dose of lithium was diminished, resulting in a concentration of about 0.5 mmol/l.

## Excellent Lithium Responders

In the previous section, we used the term “excellent lithium responders” (ER) introduced by the Canadian psychiatrist, Paul Grof (1999), for patients whom on monotherapy with lithium experienced a dramatic change in their life as their mood episodes were prevented. We compared 60 patients who started lithium prophylaxis in the 1970s, and 49 patients beginning this procedure in the 1980s. The patients were followed-up for 10 years. Those without mood episodes during this period (i.e., excellent lithium responders) made 35% of the first group and 27% of the second one. This can represent overall, roughly one-third of bipolar subjects treated longitudinally with lithium (Rybakowski et al., 2001). Grof (2010), based on his analysis of the clinical features of ER, suggests that lithium responders can be characterized by distinct mood episodes, with full remissions between them, the absence of other psychiatric morbidity and frequent history of bipolar illness in their families. This would remind the aspects of the illness, defined by Kraepelin (1899) as “manisch-depressives Irresein.”

Successful lithium therapy may favorably influence clinical, neurobiological and neurocognitive components of the progression of the illness. Such an effect is especially evident in the group of ERs. In such patients, due to complete prevention of affective recurrences, the progress of the illness is halted. A significant decrease of recurrences in partial lithium responders may also result in slowing down this process. A decrease in serum BDNF has been postulated as a marker of the later stage of bipolar disorder (Kauer-Sant’Anna et al., 2009). In our study, we have found that excellent lithium responders with a mean of 21 years of lithium treatment have normal serum BDNF levels (Rybakowski and Suwalska, 2010). We have also demonstrated that in lithium-treated patients experiencing a long-term euthymic status, the inflammatory cytokine concentrations did not differ from those observed in healthy persons (Remlinger-Molenda et al., 2012).

The magnitude of prophylactic lithium response makes a good topic for molecular-genetic studies. A review of genetic influences on the efficacy of lithium prophylaxis was made some time ago by the author of this article (Rybakowski, 2013). Recently, the first genome-wide association study of lithium response was published, in which 2,563 patients were included coming from 22 participating sites from the International Consortium of Lithium Genetics (ConLiGen). A single locus of four linked single nucleotide polymorphisms (SNPs) on chromosome 21 met genome-wide significance criteria for association with lithium response. This region contains two genes for long, non-coding RNAs (lncRNAs) which are important regulators of gene expression in the central nervous system (Hou et al., 2016).

Recently, we described five patients (two men and three women, aged 64–79 years) with ultra-long-term lithium treatment (40–45 years) and good response to such treatment. Serum lithium level was kept in them within the range of 0.60–0.65 mmol/l, except for one male, having 0.7–0.8 mmol/l. Both males had impaired renal function. However, no progression has been observed within the last 5 years. One female suffered from Hashimoto’s disease and was treated with levothyroxine. In all patients, the cognition and professional activity were at the level of healthy subjects with comparable age and education’s years. Their functioning in family and social roles was good. The beginning of lithium prophylaxis usually has been made within the first 3 years of the illness. Therefore, we could conclude that, in patients with favorable response to lithium, such a longitudinal administration of the drug can produce satisfactory performance in vocational and psychosocial areas, and the management of potential adverse effects can be adequate (Permoda-Osip et al., 2016).

## Conclusion

The evidence provided in this review article strongly suggest that negative perception of lithium as a first-line candidate for the prophylaxis of bipolar disorder can be challenged, based on the data showing its clinical efficacy and the possibility of managing its main adverse effects. There has also been a trend (see Post, 2018) to begin prophylaxis with lithium in the initial stage of the illness. Kessing et al. (2014) demonstrated that starting lithium treatment following the first manic episode is associated with increased probability of good response. There has also been data that such an approach can provide a favorable influence on the neuroprogression of the illness. Recently, it was found that, after the first episode of mania, lithium was superior to quetiapine in limiting white matter reduction and regulating neural connection between the ventral striatum and the cerebellum (Berk et al., 2017; Dandash et al., 2018).

While lithium is an orphan drug, without active supporting by any major pharmaceutical company, it only remains the sound scientific evidence which can promote the more extensive and long-term application of lithium in mood disorders, and especially, bipolar disorder. A belief can be expressed that this review may provide a small contribution to this aim. It is also hoped that the data on the neuroprotective effect of lithium contained in these articles’ collection can bring novel possibilities for the therapeutic use of the drug.

**Figure 1:**
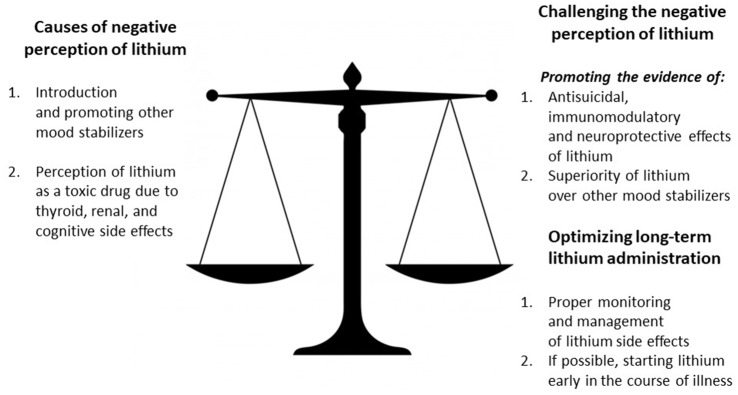
Underutilization of lithium: causes and countermeasures.

Illustration of a balance for the causes for negative perception of lithium and the countermeasures for its challenging and optimizing long-term lithium administration is summarized on Figure 1.

## Author Contributions

The author confirms being the sole contributor of this work and approved it for publication.

## Conflict of Interest Statement

The author declares that the research was conducted in the absence of any commercial or financial relationships that could be construed as a potential conflict of interest.
